# Considerations for the Retirement of Therapy Animals

**DOI:** 10.3390/ani9121100

**Published:** 2019-12-09

**Authors:** Zenithson Y. Ng, Aubrey H. Fine

**Affiliations:** 1 Department of Small Animal Clinical Sciences, University of Tennessee College of Veterinary Medicine, 2407 River Drive, Knoxville, TN 37996, USA; 2 Department of Education, CA Poly State University, 3801 W Temple Ave, Pomona, CA 91768, USA

**Keywords:** therapy animal, retirement, animal welfare, animal assisted intervention

## Abstract

**Simple Summary:**

Retirement is typically regarded as a well-deserved reward earned after a lifetime of work, but this termination of an animal’s career has potential positive and negative implications for animal, handler, and human participants in these interventions. The question of precisely when and how to appropriately retire an animal is usually answered at the discretion of the handler; however, the validity of this question remains largely unanswered without scientific evidence. The purpose of this review is to describe the implications of therapy animal retirement for the handler, participant, and animal and to discuss the challenges in determining when to retire a therapy animal.

**Abstract:**

With the growth of animal-assisted interventions, the number of animals designated to work as therapy animals continues to increase. These animals participate in this work in varying capacities during life, but there will be a point in time when the animal can no longer engage in these activities. The concept of retirement, or withdrawing the animal from its working life, is an important phase of life that every therapy animal will inevitably face. Retirement is typically regarded as a well-deserved reward earned after a lifetime of work, but this termination of an animal’s career has potential positive and negative implications for animal, handler, and human participants in these interventions. The question of precisely when and how to appropriately retire an animal is usually answered at the discretion of the handler; however, the validity of this question remains largely unanswered without scientific evidence. The purpose of this review is to describe the implications of therapy animal retirement for the handler, participant, and animal and to discuss the challenges in determining when to retire a therapy animal.

## 1. Introduction

As a result of the widespread interest and research in the human–animal bond, we have a deeper understanding of the impact of animals on human beings in society. Whether the effects of animals are due to their formal working roles as therapy animals or informally as companions, they substantiate and advance the field of animal-assisted interventions (AAI). Kruger and Serpell (2010) first defined the umbrella term AAI as “any intervention that intentionally includes or incorporates animals as part of a therapeutic or ameliorative process or milieu” [1]. AAIs encompass both animal-assisted activities (AAAs) and animal-assisted therapies (AATs). Animal-assisted activities (AAAs) are non-goal directed, informal, and often spontaneous interactions between a human participant and therapy animal facilitated by a volunteer handler to provide motivational, educational, and/or recreational benefits to enhance the quality of life of the participant. Animal-assisted therapies (AATs) are goal-directed, formal, planned, structured, and documented therapeutic interventions between a human participant and therapy animal facilitated by a health and human service provider to meet a specific beneficial goal for the participant. AAAs and AATs may be conducted with a diverse range of participants in a variety of settings, such as healthcare facilities, assisted-living facilities, and schools [2]. Although any species of animal may potentially be registered as a therapy animal, dogs are the most frequently registered and studied therapy animals [3,4,5] and will be the focus of this discussion.

There is no doubt that the popularity of therapy animal work has grown significantly in recent years. More than ever before, rigorous research is being conducted to validate the beneficial roles therapy animals have on human outcomes. Typically, AAIs are implemented to improve the welfare of humans. As we continue to increase the use of these animals, the field of AAI is obligated to scrutinize the welfare of these animals.

While the welfare of therapy animals during their working years has begun to be addressed [6,7], there has been little exploration of their welfare towards the end of and after their working careers. The idea that therapy animals should “retire” from work is a topic that has received minimal attention in the literature. AAI research, of course, focuses on the effects of actively working animals on humans, and a retired therapy animal is no longer a part of the therapeutic process. Understandably, there is little motivation to study retired therapy animals since they are no longer serving to purposely benefit humans. Nonetheless, a retired therapy animal has performed a service and should be honored for its work. Only recently has the topic of retirement of assistance animals been addressed [8]. In contrast to therapy animals that are trained to work with multiple participants under the guidance of a single handler, assistance animals are dogs or miniature horses individually trained to perform specific tasks for the benefit of only that single individual handler with a disability [9]. The retirement of an assistance animal carries separate implications and considerations from therapy animals. The working lives of assistance animals are traditionally more arduous than therapy animals because of the intense training, high skill expectation, long work duration, and exposure to the stressors of all public access environments. Therapy animals have variable training and skills that are not incumbent to an individual’s ability to function, work sessions that are not intended to be long or strenuous, and exposure to the stressors of environments only where they are permitted. Additionally, the retirement of an assistance animal may have a dramatic impact on the single individual handler because it impacts that handler’s ability to function in life, whereas the retirement of a therapy animal may have variable impact not only on the handler, but on the participants the therapy animal visits as well.

From an animal welfare perspective, retirement is a critical and necessary phase of every therapy animal’s life. The AAI field should embrace the concept that an animal may be relieved of duties if welfare is compromised during the work. This is especially true since the decision to become a therapy animal was not the animal’s choice, but rather the decision of the owner or handler. As property, the animal does not have the right or the ability to voice whether it wants to become a therapy animal and whether it wants to retire. Because the animal lacks the freedom of choice and work may be distressing, welfare should always be kept in high regard.

Although the right to retirement is inherently a positive concept in regards to animal welfare, negative implications should also be considered. The cessation of work may be emotionally distressing for the animal, handler, and/or participant that desire to continue to work. An animal that thrives on human interaction may be frustrated when a change in physical health status warrants retirement. Another ethical conundrum presents itself when the therapy animal needs to retire, but the handler is not ready to retire and the participants do not want the animal to retire. Understanding the best time to retire and how to retire gracefully may minimize the consequences of ending this work.

The retirement of a therapy animal is a complex issue that requires attention and further exploration. The purpose of this review is to define retirement in the therapy animal; to discuss the effects of retirement on the animal, handler, and participant; and to recommend when and how to best transition a therapy animal into retirement. Although the focus of this review will be on the dog because it is the most frequently used therapy animal, the concepts may be applied to any species of animal used in AAIs.

## 2. Characteristics of the Therapy Animal

Every therapy animal begins its life as a pet, also known as companion animal, defined as an animal domesticated and treated with fondness that is not required to have special training or skills [10]. As a pet, the animal is free of obligations. This freedom changes once the owner decides to designate the pet as a therapy animal. The decision to register the animal as a therapy animal is typically based on the owner’s personal desire to volunteer or work in AAIs if the animal’s calm temperament and social personality are fitting to be a therapy animal. The owner will meet the requirements to be registered as a therapy animal by a therapy animal organization. Depending on the individual therapy animal organization, these requirements may include handler training and testing, animal training, veterinary evaluation, and behavior evaluation [11]. Typically, the owner serves as the handler of his or her animal; a person other than the primary owner may be trained and registered to handle the animal as a team.

To qualify as a therapy animal, the animal is expected to behave a certain way while working. Equipped with basic obedience skills, these animals are expected to be reliable, predictable and controllable [12]. The therapy animal working in AATs may be expected to perform at a higher level of obedience when compared to AAAs since the handler is a health and human service professional working with the animal as a part of his or her profession. The animal should be inherently gentle and placid while possessing an affiliative nature with a strong desire to interact directly with unfamiliar humans [13]. This may be demonstrated by a therapy dog’s extended gaze into the eyes of a human [14]. A behavior evaluation screening for therapy animals should monitor for excessive indicators of stress in the animal and conclude that there is no concern for the safety or welfare of the animal or participants [2]. Mature dogs are selected as therapy animals more often than young (less than two years of age) dogs because they are calmer and demonstrate fewer behavioral indicators of stress [15]. Although some recipients have remarked that short haired dogs of medium size are preferred therapy dogs [13], any breed of dog has the potential to be a therapy animal. Since there is significant variation in standards among therapy animal organizations and there is a lack of descriptions of the qualities of and training of animals used in AAI research [5], it is challenging to capture evidence-based guidelines for the ideal therapy animal. Ultimately, the temperament of the animal is more important than the physical traits of the animal in becoming a proper therapy animal [16].

It is important to remember that the therapy animal does not retain this designated role at all times. When the therapy animal is not working, it is considered a pet, which is free from working obligations. The amount of time the animal works as a therapy animal versus lives as a pet is typically at the discretion of the handler.

## 3. Evidence That Therapy Work May Be Stressful

Positive human–animal interactions should be beneficial for both humans and animals alike [17]. Under most circumstances, people perceive the animal to enjoy the interaction. This may hold true, as there is evidence that affiliative biomarkers increase in dogs after human interaction [18]. Handlers often remark that the animal is excited to engage in work.

Overall, there is little evidence that therapy work is significantly stressful if the appropriate animal is chosen, handlers are educated, and the intervention is not forced [6,19,20]. However, some therapy animals may not necessarily benefit from these interactions, but rather obediently tolerate these forced interactions. The animals that endure stress without displaying obvious signs of stress may in fact be negatively impacted. Some factors that may influence the stress to the animal may include the type of people visited, the environments there, the quality and quantity of interactions, and the handler themselves [20]. When these factors are balanced with the welfare of the animal in mind, positive interactions can occur. Even for animals that enjoy the interactions, there may be a threshold, whether it be in duration, frequency, or intensity, at which the animal tires and the interaction is no longer beneficial and perhaps even be distressing to the animal.

As animals age, they are less resilient to change and have more difficulty recovering from stressful events. Studies have found that older dogs show less interest in interacting with strangers and are less able to cope with social distress than younger dogs [21]. In one study, aged dogs behaved more passively, showed less interest in an unknown person during separation from owners, and had a significantly increased stress response. This indicates that older dogs may not be as adept at managing social situations, especially since age decreases the sociability of the dog [22]. Interestingly, aging dogs also transition from spending a lot of time directly interacting with humans to simply spending more time near humans [23]. Therefore, a handler should not be surprised that an older therapy dog may choose simply to be around, but not necessarily engage with the handler. Retirement from the social challenges encountered during assistance animal work can be beneficial to the welfare of these dogs.

Because of the potential negative implications of the work, a therapy animal should not actively work until the end of its life. Typically, aging dogs develop conditions that prevent their ability to successfully fulfill their working duties. However, there have been a few reports of dogs working until their dying days. One study reported that a therapy dog working at a children’s hospital “died peacefully in her home in the care of her handler” prior to the conclusion of the study [24]. Another study conducted at a children’s hospital reported “one elderly AAT dog…died before her next scheduled shift” [15]. Details regarding the cause of death are unknown, but beg the question of why these dogs were working until death and not retired in advance. Unless the cause of death was a sudden, unforeseen accident, these dogs should have been removed from work if any signs of illness were observed. While it is unlikely that the therapy work significantly contributed to the cause of death, the conditions of the animal’s work could have been distressing to the dog and impaired its ability to recover from the illness that eventually ended its life. This was proposed in another study describing the stress endured by a single dog working frequently with severely disabled children who tried to deliberately injure him [25]. This added stress may have been associated with this dog’s chronically elevated cortisol levels and associated disease state [25]. Similarly, another study implicated that intense therapy work resulted in behavioral problems in a therapy dog that led to its eventual retirement from work [26].

These are examples from research reports that volunteered to reveal these outcomes, but these adverse events may occur more often than we know. The precise number of therapy dogs that work without a definitive period of retirement before death is unknown. Although these circumstances likely occur infrequently, the death of a therapy animal that was not granted an appropriate period of retirement should nonetheless still be regarded as a serious issue. Because of the potential stressors of therapy work, it is important that the animal be retired from these activities so as not to negatively impact welfare. Therefore, it is important to consider retiring a dog from any working condition long before it is negatively impactful to its welfare and health status.

The process of retirement can be a significant lifestyle change for an animal, just like for a human. Unfortunately, handlers may continue working therapy animals under conditions where they appeared fatigued, uninterested, or not completely fit either psychologically or physically for the tasks with which they were involved. Regrettably, when these situations are witnessed, some do not take the moral responsibility to counsel the handler about the problem and encourage said handler to alter an animal’s visitation schedule or consider retirement. Both of these outcomes would support the animal’s best interest.

## 4. Defining Therapy Animal Retirement

Retirement, the withdrawal from one’s position, career, or working life, is a human concept that stems from the desire and ability to be free of work obligations. This topic is of upmost importance in today’s world because of technological advancements in medicine, permitting humans (and animals) to live and sustain healthier and longer lives than ever before [27]. Of course, longer lives lead to longer retirement [27]. There are similarities and differences when comparing retirement between people and therapy animals.

Logically, freedom from work obligations is perceived to be a positive reward, frequently celebrated by people. The transition from worker to retiree allows the individual to enjoy leisure time occupied by hobbies, volunteerism, travel, and rest [28]. These retirees have the freedom to choose how they spend their time. More leisure time for the retired therapy animal may involve more rest. However, the amount of rest is determined by the owner, so the animal is not afforded the same freedoms as humans. On the other hand, retirement is a life phase for which a person anticipates and plans. A therapy animal does not have the cognitive ability to conceptualize or expect retirement; every activity is at the will of the owner because an animal is legally and ethically considered property [29].

People may retire if health ailments prevent them from work or if they reach a certain age. If a worker maintains good health in old age, retiring at 65 years of age has been recommended because this is the age at which cognitive decline may reduce workplace productivity [30]. Similarly, animals that are expected to be active and engaged during work may not perform as well due to the cognitive decline associated with aging. However, many therapy animals still generate positive benefits for human participants in AAAs and AATs by merely being present and passive. While an employer may be financially motivated to retire an underperforming worker, a therapy animal organization does not have a similar incentive to retire a therapy animal. In fact, the retirement of a therapy animal represents a loss for the organization.

The critical difference between retirement in people and therapy animals is the ability to enter retirement, which is ultimately dependent on the person’s financial independence [31]. The person can elect retirement once he or she has saved enough money during his or her career to be able to maintain a lifestyle suitable for the individual. The benefit of a pension may also dictate whether a person is financially able to retire. If financial independence is not achieved, the person may be obliged to work past retirement age. Since therapy animals are not paid employees, this is not a factor for their retirement. Rather, the main reason to retire a therapy animal is to guarantee good welfare by ensuring the animal is not forced to work when it does not want to or is unable to work. As opposed to people who must work to earn a living, there is no reason an animal *must* work.

There are two methods of retirement for a therapy animal: full or semi-retirement. Full retirement is the animal’s complete and final withdrawal from AAAs or AATs. Semi-retirement is a reduction in frequency and/or duration from AAAs or AATs before the animal is fully withdrawn from the work. This may include transitioning to work with fewer participants per session, different populations, or different settings where the workload may not be as taxing. The decision to enter full retirement or semi-retirement, the manner in which semi-retirement is executed, and the length of transition from semi- to full retirement depends on the reason for retirement in conjunction with the animal’s response to AAAs and AATs. In retirement, the retired therapy animal returns to function as a pet.

## 5. Implications of Retirement

Retirement represents one of the greatest life milestones one can experience. It is bittersweet because, while it represents the end of a career, it marks the beginning of freedom. Because of the intimate nature of therapy animal work, unique relationships and bonds form between animal and handler, as well as animal and participant. Each member of this triad is significantly impacted by the retirement of the therapy animal. The permanent cessation of work carries both positive and negative consequences and considerations for the therapy animal, the handler, and the participants.

### 5.1. For the Animal

The effects of retirement on the animal itself are central to the decision to retire a therapy animal. As previously stated, the benefit and main reason to consider retirement is to ensure welfare by not forcing an animal to work when it does not want to work. The freedom from work safeguards the animal from the stressors associated with AAAs and AATs, such as long and frequent interventions, negative interactions, and exposure to infectious disease [32]. It is clear that retirement is beneficial for the animal that demonstrates signs of stress and aversion to human interaction. However, it is particularly important for the animal that does not demonstrate these signs clearly, but does not enjoy its role as a therapy animal. Because of their obedient nature and desire to please their owners, therapy animals may not display obvious behavioral signs that they do not enjoy the interactions. Instead, they tolerate the AAAs and AATs without the handler being aware that the work is distressing and negatively affecting the animal’s welfare state. Removing the animal through retirement assures that these obedient therapy animals are not forced to work against their wills. If the animal is retired because of illness, retirement gives the animal the opportunity to recover without being subjected to the stress of human interaction and that of therapy work. Moreover, common physical ailments that increase with age, such as osteoarthritis, may be overlooked or attributed to the dog “just getting old,” as therapy work continues [33]. Since the activity associated with some types of AAAs and AATs can exacerbate osteoarthritis and other chronic diseases, retirement allows freedom from these added stressors. Retirement also permits adequate periods of rest, which is essential to the health and welfare of the aging animal [34]. The golden years of retirement should ideally be spent in quiet relaxation.

Retirement may not always be restful or beneficial for the therapy animal, however. The resort to a sedentary lifestyle may result in boredom or frustration for the animal that needs to have a purpose and job in life. In addition, physical and mental stimulation retard the aging process in dogs [35,36,37]. Specifically, social interactions constitute a critical component of healthy aging [38]. The physical and mental stimulation associated with AAI, especially play, may be beneficial for the animal [39]. Retirement and, thus, the discontinuation of this stimulation may expedite the aging process, especially if therapy work is not substituted with other stimulating and engaging activities. Those animals that thrive on and desire human interaction may want to work beyond their physical limits.

The bond between handler and animal may be challenged when retirement occurs by virtue of the fact that the amount of time spent together is decreased. If the handler leaves the animal behind when he or she would typically take the animal to an AAA or AAT, the animal may become frustrated at being left behind, not understanding why [40]. In addition, this abandoning act may result in separation anxiety and subsequent adverse behaviors in the animal, especially the highly attached therapy animal that spent a significant amount of time with the handler [41,42]. Animals typically grow accustomed to a routine, especially one where therapy sessions are conducted frequently and regularly [42]. When the animal retires, the sudden, drastic change in routine may be difficult for the animal to adapt to, which may be a reason to elect semi-retirement rather than full retirement.

### 5.2. For the Handler

While retirement of the therapy animal certainly changes its lifestyle, it may have more significant effects on the handler. The impact of therapy animal retirement on the handler depends on the handler’s perception of animal welfare and personal motivation for participating in AAAs and AATs. The handler will view retirement as a positive experience if he or she has a strong desire to safeguard welfare and wants what is best for the animal. With retirement, the handler does not have to worry about continuing to work the animal past its limits or subjecting it to work stressors. In addition, retiring the therapy animal allows the handler to not be plagued with potential guilt if the animal works until its dying day; the handler may feel responsible for not providing the animal the proper rest and respite before its death. Retiring the animal also means that the handler is free of AAA and AAT responsibilities, which is a relief for the handler that no longer wants to participate or is overburdened by the work. Some handlers may be ready to stop volunteering, and the animal’s retirement may be perceived as a welcome opportunity for a natural ending. Retiring the animal does not negate the fact that the animal is still the handler’s companion, and they can still engage in other enjoyable activities together.

However, the retirement of the therapy animal may prove to be a difficult process to navigate and accept for the majority of handlers. This is largely because of their motives to continue volunteering. People strongly relish having leisure time to pursue various activities, especially volunteer work [43]. Many registered therapy animal handlers have this leisure time because they are retired themselves. There are many health, psychological, and social benefits derived from volunteerism, including fulfilling altruistic needs and attaining a sense of self-satisfaction, especially in mid-life and older adults [44]. Each individual will have personal motivations for volunteering that aligns with enjoyment and satisfaction [45]. Handlers often want to give back to their community, help others, or have a hobby [46]. Considering that volunteering alone is beneficial, volunteering with one’s personal pet may enhance the benefits to a greater degree. Individuals who can connect their passion for their animals alongside their volunteer duties may find the volunteer work to be more rewarding and even more meaningful to an individual than other volunteer activities.

Retirement represents not only the loss of volunteer benefits and activity to fill leisure time, but also the loss of identity and purpose. Handlers maintain an identity as a team with their animals, and upon retirement, they are no longer a team. In addition, the handler is typically highly attached to the animal. Retirement reduces the time spent together, which can be difficult for the handler to cope with. Teams of handlers and therapy animals are proud to uphold the purpose to improve the lives of those with which they interact. Many volunteers may feel that the participants are depending on them. Retirement may bring the burden of guilt that handlers are no longer helping the participants with which they have previously engaged.

A therapy animal organization strives to maintain and increase numbers of active volunteer teams, which makes retirement counterproductive. Davis (2003) reports that one of the major attributes of engaging volunteers is to fulfill their need to still be wanted and recognized for their contributions [47]. The handler may be compelled to continue work because the therapy animal organization provides rewards and accolades for the amount of work achieved. Handlers may aim to reach a minimum number of volunteer hours, which may obscure their acceptance of retirement. The volunteer organization may feed the handler’s obligations to fulfill his/her needs, meet hour requirements, and achieve rewards and accolades for work. Although it is more common for therapy animals to participate in AAAs with volunteers, they also participate in AATs with professionals. These professionals earn a living by using therapy animals in therapeutic plans. The financial and professional impact associated with the loss of working with an animal may cause a professional to be reluctant to retire an animal when he/she should.

Finally, the handler may find it difficult to consider retirement because it foreshadows the imminent mortality of the beloved animal, which is upsetting. These considerations make a handler less willing to realize or accept retirement of the therapy animal. However, the handler that does not allow his/her personal motivations to cloud his or her perception of animal welfare will advocate retirement when appropriate. Regardless of these considerations, it would be dangerous and unethical for a handler to put his or her own feelings before the animal’s welfare, denying the retirement it deserves.

### 5.3. For the Human Participants

It is critical when retiring a therapy animal to recognize the impact of the therapy animal’s retirement on the participants served. Many clients develop significant bonds and attachments with therapy animals they see regularly, where they develop rich relationships and strong bonds [48,49]. They become personally attached to that particular animal and look forward to future visits [50]. Retirement can be difficult for the participant to accept, especially when they benefit from the interventions. Even if the therapy animal is replaced with a new therapy animal, the participant may have unmet expectations because of the accustomed personality and demeanor of the previous animal. If the retired therapy animal is not replaced, the animal’s retirement may also denote withdrawal of a particular handler with whom the participant has developed a relationship. The complete loss of the team can certainly impact and be detrimental to the participant’s wellbeing. The discontinuation of AAA and AAT could leave a sense of abandonment and loss that not only prohibits any potential future benefits from the human-animal interaction, but may even impact or reverse the progress that was made through therapy [51]. The stronger the relationship is with the animal, the more difficult the experience could become [52].

However, if retirement is approached strategically, it may be well received by the participant. The participant likely cares for the animal and wants what is in the animal’s best interest. When retirement is discussed in the context of welfare, the participant may be more accepting of an anticipated outcome that is perceived to be difficult. In addition, engaging in AAAs and AATs with an aging or geriatric animal that should be retired may be difficult for a participant. The therapy animal may appear weak, disengaged, and sick, negating the mutually beneficial effects normally achieved with a healthy and robust animal. Clients may feel fear, unease, and pain waiting to see if the animal gets better [51]. Appropriate retirement prevents this from occurring. In addition, appropriate retirement minimizes the chance of an acute and sudden death of the animal. Informing a participant that a therapy animal has passed away is tragic and unsettling, especially since the participant was not prepared to say goodbye to the animal before death [51,53]. Retirement facilitates a peaceful goodbye and closure for the participant.

## 6. When to Retire a Therapy Animal

Currently, there are no evidence-based studies indicating the ideal time for therapy animal retirement. Determining when a therapy animal should retire is unclear, multifactorial, and dependent on the individual. Just as the animal does not decide out of its own volition to become a therapy animal, the animal cannot voice when it wants to retire. This decision should be based on careful assessment by the handler in conjunction with a veterinarian and/or behaviorist familiar with the animal and the duties, working conditions, and potential stressors of that particular type of therapy work. Similar to assistance and working dogs, consideration for retirement may be advised based on age, physical health, and behavior [54,55,56,57,58]. As discussed, the animal, handler, and participant are each impacted by this significant change in different ways, but the welfare of the animal should be the highest priority when deciding to retire the animal.

It is reasonable to consider retiring a therapy animal at a targeted age, similar to the way people typically retire at 62–65 years of age. However, it is challenging to set a universal and specific age cut-off because of the variability among breeds of dogs, let alone species of animals. It has been recommended that retirement be considered for assistance dogs once they reach the senior life stage for that particular breed, typically between 10–12 years of age for many working dogs of large breeds [56]. This ensures that the assistance dog, who has worked and was potentially on-call every day since puppyhood, can enjoy a well-deserved retirement while in good health. The average therapy dog working for one hour once a week for a few years has not worked at the same capacity as other working dogs. Furthermore, assistance dogs typically enter the workforce at a very young age because they were specifically bred for that role, whereas most therapy dogs enter the workforce at an older age because mature dogs are more calm and amenable to therapy work. The late entrance into the workforce shortens the duration of working life compared to assistance dogs, making age less of a critical determinant for retirement in therapy dogs.

Although age is only a number and a crude manner of dictating retirement, it indicates when a handler should be more vigilant about monitoring the animal for health ailments. Once a therapy animal does reach a senior life stage as defined as the last 25% of life expectancy [59], comprehensive veterinary evaluations and diagnostics, such as complete blood count, serum chemistry, urinalysis, and thyroid level, should be monitored at six-month intervals to screen for subclinical disease and implement necessary preventive care. Many health conditions, such as cancer, progress asymptomatically until they have substantially compromised affected organ systems. Detecting these diseases earlier by bringing the aging animal in for regular evaluations may permit earlier treatment and better prognoses. Of course, the diagnosis of a subclinical disease may warrant temporary suspension or permanent retirement from therapy work to minimize additional stress on the body.

Significant changes in health and behavior will dictate the appropriate time to remove an animal from work either temporarily or permanently regardless of age. If the animal succumbs to acute illness, the work should be postponed until the animal returns to normal health. If the acute illness persists chronically (longer than one month) and is not controlled or undiagnosed, permanent retirement should be considered. Certain conditions that may worsen with the stimulation and activity of therapy work require retirement. Pain associated with musculoskeletal disorders, such as osteoarthritis, may increase in AAI sessions that warrant movement or increased weight bearing. Difficulty breathing and persistent coughing associated with cardiorespiratory conditions may be exacerbated with increased activity in AAI. Seizures and other unpredictable neurological conditions may be increased with the stimulation of AAI. Stress colitis may also worsen if the animal is stressed during the work. In addition, an animal with diseases consisting of vomiting, diarrhea, increased urination, urinary incontinence, and fecal incontinence may make an inappropriate therapy animal. These eliminations all present a hazard to the people involved, along with distressing the animal. Participants in AAI are also likely to be distressed by seeing an animal in pain, having difficulty walking or breathing, and/or having seizures. Efforts should be made to avoid these scenarios by removing the animal from work at the first indication of clinical illness.

Not all diseases or conditions necessarily warrant the retirement of the therapy animal. There are various chronic disease conditions a therapy animal may be diagnosed with that do not require the animal to retire if the condition is clinically well managed, free of symptoms or pain, and the animal is otherwise deemed healthy for all intents and purposes. Of course, if the animal is newly diagnosed with the condition or demonstrating clinical symptoms despite receiving therapeutic interventions to manage it, visits should be suspended. While we recommend that therapy dogs experience good health during retirement, retiring an animal while it is completely free of disease may not necessarily be mandated, especially for those that appear to enjoy this leisure activity. If the disease is not exacerbated by therapy work, the animal may continue to work without detriment to welfare. For example, endocrine conditions frequently diagnosed in older dogs, such as diabetes or hypothyroidism, may be well controlled with medications. If the condition does not impair the animal’s ability to work and is not worsened with the stress and stimulation of therapy work, the animal may continue working. However, if the animal is on treatments that may present a risk to humans, such as chemotherapy or antibiotics, the animal must be removed from therapy work until treatments are completed and the veterinarian has given clearance to return to work. The decision to continue work and to make modifications, such as a reduction in frequency, duration, or intensity should be done on a case-by-case basis in conjunction with the veterinarian.

Behavioral assessment is the other critical component to determine when an animal should retire. The animal may display subtle signs indicating it does not want to participate in AAI. These may include increased restlessness, snout licking, paw lifting, yawning, body shaking, nosing, circling, increased locomotor activity, and lowering of body posture [60]. Often, there are specific triggers that predict a therapy visit, such as putting on a bandana, harness, grooming, or specific verbal cue. The animal should exhibit excitement upon recognition of these triggers at the anticipation of a therapy session. However, if the animal retreats, hides, or demonstrates stress-associated behaviors with these anticipatory cues, retirement should be considered. During the visit, the animal may exhibit abnormal behaviors suggesting that the animal does not want to work, like resistance to entering the facility or rooms, avoiding human interaction, not looking at or engaging with participants, and ignoring commands. Given the docile and obedient nature of therapy dogs trained and registered for work, a stressed therapy dog is less likely to react dramatically or aggressively compared to another dog. They may continue to tolerate the work because it is forced upon them by the handler, but not necessarily enjoy human interaction, which is manifested through subtle stress-associated behaviors. In more severe circumstances, “learned helplessness” may be witnessed when an animal struggles to avoid an aversive stimulus (human interaction) it is repeatedly subjected to, but cannot escape, so it surrenders as if its helpless to change the situation [61]. An animal that shows signs of resistance or subsequent defeat because the handler forces it to interact with a human being is unfit to be a therapy animal. This demonstrated form of “learned helplessness” is detrimental to welfare and may ultimately result in an adverse event if not addressed [61]. After the visit, the dog that is ready for retirement may not recover appropriately and appear lethargic, listless, and exhausted for an extended period of time after the session.

If these abnormal behaviors occur once and do not recur after future sessions, the dog may have had an off day or was reactive to the particular situation. However, if these signs are observed on subsequent visits, a veterinary consultation is recommended. These behavioral changes may be a result of a physical ailment or the work distressing the animal. If the animal is deemed healthy, behavior consultation and re-evaluation is recommended. If the same adverse behaviors occur in other therapy facilities, people, and contexts, retirement should be considered.

The handler’s role as the animal’s advocate is of utmost importance. The handler is most attuned to the animal’s needs and any changes in attitude or behavior. Stress can be challenging to detect, especially if the behaviors are subtle or change gradually. Therefore, the handler should be equipped with the education and training to recognize behavioral indicators of stress, especially those that have been established in dogs such as lip licking, yawning, panting, paw lifting, body shaking, and lowered posture [60]. Because stress behaviors are highly variable from dog to dog, the handler should intimately understand the individual animal’s normal behavior and implement proper protocols when changes or adverse events occur. With the guidance of a behaviorist and/or veterinarian, a proper plan to evaluate the animal and modify the work or suspend work can be implemented to ensure its welfare. The maintenance of the therapy animal’s welfare begins with a perceptive and objective handler. As discussed earlier, a handler may be blind to stress signals when there is a personal motive to continue volunteering.

When personal bias is in question and handler objectivity in assessing welfare is difficult, quantitative surveys may be revealing. A survey on therapy dog quality of life (QoL) may be a tool to assist the handler when considering retirement for an aging animal free of clinical disease or pain. Many QoL surveys and scales have been developed to assist pet owners in deciding when to euthanize an animal [62,63], but no scale has been established for deciding to retire therapy animals. The proposed scale for assistance dog QoL (Table S1) requires the handler to objectively assess 10 factors that may be indicative of the animal’s current welfare state: sociability, enthusiasm for work, playfulness, energy level, rest, mobility, appetite, predictable eliminations, obedience, and minimal displays of stress. Any animal with chronic disease or pain should be relieved of work duties. The survey should be taken while the animal is in optimal working capacity to provide a baseline score and then retaken when retirement is in question. Since QoL is specific to the individual rather than using a universal cutoff value, a decreased score of more than 25% from baseline warrants the consideration for retirement in conjunction with veterinary and behavioral consultation. These instruments should not be used in isolation, but rather to begin a conversation with a veterinarian, behaviorist, or other animal expert [64], as this instrument is intended to detect subtle declines in QoL.

## 7. How to Retire a Therapy Animal

The transition from working life into retirement must be executed carefully to minimize the potential consequences to all parties. Two keys to successful retirement require an adjustment to the loss and social ties of work and a satisfactory retirement lifestyle [65]. Retirement should be facilitated by the organization under which the therapy animal works. However, formal policies on when and how to retire and what to expect during retirement are not traditionally addressed by most organizations. Therefore, handlers are often unprepared or unequipped to navigate the challenges associated with retirement. To conquer these challenges, organizations should educate handlers about the issue early in the animal’s career and counsel them once the animal reaches this stage. Guidelines should inform the method of transitioning into retirement that balances the welfare and wellbeing considerations for the animal, handler, and participant.

### 7.1. Considering the Animal

The transition to retirement for the therapy animal simply means that the animal exclusively assumes the role of a pet or companion animal. At first glance, the transition appears rather simple and benign. However, abrupt changes in routine may induce stress in the animal. How to properly retire an animal depends on the extent of involvement in interventions. An abrupt end to work for a healthy animal may be a negligible change. However, for an animal that works frequently for long periods of time, an abrupt end may be a dramatic change, breaking a routine and expected activity in the animal’s life. Those that participate with less frequency or duration may be less affected by a cessation of activities. The complete discontinuation of therapy works for an ill animal or animal that visits less frequently (less than once a week) may be discontinued indefinitely, likely without much consequence as long as the other needs are met.

The animal may transition into retirement by entering semi-retirement, which involves working at a reduced capacity prior to complete cessation of work duties. The AAAs or AATs may be reduced in duration, frequency, and/or intensity. Extending intervals between sessions by 25% or reducing the duration of sessions by 25% every 2 weeks may gradually allow the animal to adapt to withdrawing from work. The intensity can change from more active sessions, characterized by numerous individuals directly touching the animal, to a more passive role, such as sitting in the presence of a human.

For the dog that truly enjoys human interaction, opportunities for social engagement and stimulation with people should be offered whenever possible. These opportunities may be provided by bringing other people to the home or taking the animal to engage in social situations, such as dog parks. Being naturally social, the retired therapy animal should not be isolated during retirement. In addition, the animal should continue to be stimulated with regular and frequent activities, such as play and exercise appropriate to the animal’s physical capacity. The activities and social interactions that replace AAAs and AATs require quality time between owner and animal that enhances the human–animal bond. When the owner leaves the animal at home alone, the animal should be provided with interactive toys and environmental enrichment to occupy its time. This is particularly important when the owner leaves the animal behind when it would normally be taken to work.

During the transition to retirement, the animal must be closely observed for changes in behavior that indicate the dog is distressed and can no longer work. Behavior changes, such as vocalizing, restlessness, or destruction, may indicate anxiety and frustration. Signs of separation anxiety may intensify, especially when the handler leaves the animal behind. Opportunities for social engagement and mental stimulation should be offered outside of the therapy context, as previously discussed. If the animal still displays adverse behavioral signs with these modified activities, the animal may still be used in semi-retirement at a reduced capacity. If behavior issues subside and the animal appears more content to continue working, full retirement may be detrimental to animal welfare. The decision to return to work should be made in conjunction with the therapy animal organization and veterinarian or behaviorist.

### 7.2. Considering the Handler

When retirement is determined by the animal’s need to stop working rather than the handler’s non-compulsory decision, the transition may be difficult for a handler to accept. The organization through which the therapy animal is registered should be proactive in supporting the handler during this period. The handler should be assured that retirement is the right decision and is respected by the organization and participants because it is what is best for the animal.

Once retirement has been decided upon, the therapy team should be acknowledged and retirement should be celebrated at the last visit. This can be a formal party or ceremony that commemorates and honors the retiree [66]. This is especially important for those therapy teams that have volunteered extensively at a particular facility, developed deep relationships with the regular participants or staff at a facility, or have gone above and beyond the call of duty. It serves as a formal opportunity for positive closure and for the team to be valued and recognized for its efforts. This demonstrated appreciation also makes the handler more willing to continue to volunteer in a similar or different capacity after the animal retires.

The transition to retirement may be most difficult for the handler who finds meaningful purpose in AAA and AAT and wants to only volunteer in this capacity. The most logical transition for this volunteer handler is training and registering with a new dog. However, the handler should not expect the work will be the same. The handler may be disappointed to see the new animal not perform as well as the previous one. Alternatively, the handler may still conduct AAA and AAT sessions by handling another trained therapy animal owned by a different owner. This new animal–handler team will have a different working relationship than the previous partnership. Therefore, the team should be evaluated together to ensure skill, safety, and efficacy. If the same animal is used by different handlers, scheduling should be considered to not overwork the dog.

If the motive for volunteering is to support the AAI organization, the handler may continue volunteering with the organization in a different capacity. Roles such as fundraising, clerical work, and assistance during special events, are often available for non-profit organizations. If the motive for volunteering is working with animals, other animal organizations take volunteers, such as local animal shelters. If the reason is to volunteer for the people that were visited, opportunities to volunteer directly at those facilities and engage with the same participants may be considered. Some volunteers may not have any issues with retiring. If the motive to volunteer was to spend quality time with the animal, then retirement permits the retired team to spend quality time bonding and engaging in less strenuous activities.

For those handlers who have a commitment to titles and may be resistant to demoting their animal back exclusively to a “pet,” these animals can be retitled as a “retired therapy animal.” It is important that society respect these animals for the work they have done.

### 7.3. Considering the Participant

While the transition to retirement may be difficult for both animal and handler, the participants who are accustomed to regular visits and who have grown attached to the animal may have a difficult time adjusting to the animal’s absence. The handler should inform the participant in person that the animal will no longer be visiting. It is important that the individual receive the news directly from the handler. Delivering the news in person allows for a dialogue and a time to ask questions regarding the reasons for retirement. When the reason is explained in terms of the animal’s welfare and wanting what is best for the animal, most participants will accept this outcome. In addition, the discussion of retirement stimulates conversations around empathy, limitations, wellness, loss, and resilience. These and numerous other lessons may be valuable, particularly for participants where selflessness and self-reflection are goals.

Regarding appropriate timing, the participant should also be informed of the retirement prior to the last visit. This gives the participant the opportunity to ruminate over the news and prepare a formal goodbye on the last visit. Preparation and coping with retirement may be encouraged by asking the participant to write a letter or send a gift for the animal. The last visit should be a celebration for retirement and an opportunity to show gratitude for the work the animal has done. Keepsakes, such as photos of the animal or farewell greeting cards from the team, can be given to participants. This facilitates a more peaceful closure and happy parting.

A plan should also be in place to substitute the therapy team with a new therapy team. It is important that the participant be counseled to not expect the new animal to behave or engage in the same manner as the old one. Alternatively, if a new team cannot be substituted, other activities may occupy the time previously dedicated to AAI. Activities involving animals, such as movies or visits to zoos, farms, or shelters, may be desired to avoid sudden cessation of all animal contact.

If both handler and animal retire and the participant is highly attached, occasional visits from the handler may be welcome surprises. The handler may relay stories or photos of how the animal is enjoying retirement.

## 8. Conclusions

Issues that pertain to animal welfare and preserving the quality of life of therapy animals have become more appreciated in recent years. However, retirement has been given little attention in regard to welfare. The ending of a therapy animal’s career is inevitable and can be challenging to navigate. Retirement has both positive and negative implications for the handler, participant, and animal. Although legitimate considerations to resist retirement exist, welfare must always be prioritized. Knowing when and how to retire a therapy animal properly can be difficult. Therapy animal organizations should provide training and oversight to handlers and professionals, emphasizing the handler’s role as a champion of animal welfare. Handler guidelines should explain that the handler’s utmost responsibility is to consider both the participant’s needs and the therapy animal’s needs before, during, and after work. This includes the animal’s need for retirement. Further research is necessary to address the effects of optimal methods of approaching therapy animal retirement. Mahatma Gandhi once noted, “The greatness of a nation and its moral progress can be judged by the way its animals are treated.” As the field of animal-assisted interventions continues to flourish, it is our moral and ethical responsibility to assure the welfare and wellbeing of the animals from beginning to end.

## Supplementary Materials

The following are available online at https://www.mdpi.com/2076-2615/9/12/1100/s1, Table S1: Therapy Animal Quality of Life Scale.
