# Supplementary material for: Considerations for the Retirement of Therapy Animals
Source: Animals (Basel). 2019 Dec 9;9(12):1100. doi: 10.3390/ani9121100 (PMC6941057; doi:10.3390/ani9121100)
Supplement: Supplementary file 1 [file animals-09-01100-s001.pdf]

Supplementary Materials

Table S1: Therapy Animal Quality of Life Scale

Place a check mark for what is most accurate for your therapy animal at the current moment. The healthy animal should be free of clinical disease or pain.

	Strongly Disagree	Disagree	Neutral	Agree	Strongly Agree	
**Sociability**<br>The animal is engaged, seeks attention, and stays engaged with people						
**Enthusiasm for work**<br>The animal appears excited to travel, wear any working apparel/equipment, or observe any cues that he/she is going to work						
**Playfulness**<br>The animal spontaneously engages in play						
**Energy level**<br>The animal exhibits an appropriate level of energy for the activity he/she is engaging in						
**Rest**<br>The animal sleeps with ease and appears alert and well rested when awake						
**Mobility**<br>The animal walks, runs, jumps, rises, and lies down with ease						
**Appetite**<br>The animal has a regular and consistent appetite						
**Predictable eliminations**<br>The animal urinates and defecates when expected without accidents or incontinence						
**Obedience**<br>The animal responds to commands immediately and consistently						
**Minimal displays of stress signals**<br>The animal does not exhibit signs of stress (ie., excessive lip licking, yawning, pacing, crouching, circling, whale eye, paw lifting) during work or rest						
**Total number of checks**						
**Multiply by**	0	2.5	5	7.5	10	**Total**
**Total**	+	+	+	+	=	

The survey should be taken while the animal is in optimal working capacity to provide a baseline score and then retaken when retirement is in question. The maximum total score is 100. A decrease of 25% or more from baseline score warrants consideration for cessation of work and retirement.
